# Impact of risk and lifestyle factors on therapy goals in the treatment of breast cancer and gynecological cancer patients with integrative medicine

**DOI:** 10.1007/s00404-025-08002-w

**Published:** 2025-04-09

**Authors:** Katharina Seitz, Anna-Katharin Theuser, Sophia Antoniadis, Matthias W. Beckmann, Milena Beierlein, L. Brückner, Katharina Au, Carolin C. Hack

**Affiliations:** 1 Department of Gynecology and Obstetrics, Erlangen University Hospital, Comprehensive Cancer Center Erlangen–European Metropolitan Area Nuernberg (CCC ER-EMN), Friedrich Alexander University of Erlangen–Nuernberg, Universitätsstrasse 21–23, 91054 Erlangen, Germany; 2 Institute for Women’S Health/Institut Fuer Frauengesundheit Gmbh, Erlangen, Germany; 3 Bavarian Cancer Research Center, BZKF, Erlangen, Deutschland

**Keywords:** Cancer, Gynecological oncology, Lifestyle, Integrative medicine, Complementary and alternative medicine

## Abstract

**Background:**

As a result of advancements in the diagnosis and therapy of cancer, the prognosis for cancer patients has significantly improved. The benefits of a significantly enhanced survival time lead to a more extensive concern with quality of life and managing the side effects during oncological treatment. Implementing integrative medicine strategies has been found to reduce the side effects of therapy and disease. In 2021 the S3 guideline on complementary medicine in oncology was published for the first time, which takes a stand on the most common aspects of complementary and integrative medicine in Germany. The aim was to see whether a previous healthy life style impacts the success of integrative medicine for patients.

**Methods:**

Within the framework of a cross-sectional study over 15 months, 120 cancer patients were monitored at a standardized integrative medicine consultancy service at the University Integrative Medicine Center of the University Hospital Erlangen, Department of Gynecology and Obstetrics. The basic questionnaire consisted of questions on socioeconomic background information, lifestyle factors, such as dietary habits or smoking behavior, as well as information on the gynecological situation. Furthermore, an evaluation based on patient-reported therapy goals concerning the reduction of side effects of conventional cancer treatments, enhancement of disease-related quality of life and better stress and disease management, active participation in cancer treatments, mind–body stabilization, and improvements in coping strategies were assessed. In addition, the impact of patient characteristics and lifestyle on the subjective achievement of these outcomes was evaluated to set the answers in context and show its influence. Statistical analysis was performed using SPSS Statistics for Windows version 26 (IBM Corporation, Armonk in New York, USA). Mean, standard deviation, minimum, and maximum were calculated for age and BMI. The other characteristics regarding demographics, lifestyle, tumor disease, and therapy were analyzed based on their respective absolute and relative frequencies.

**Results:**

A large majority of the patients' participation goal was to reduce cancer-related side effects (90.8%), second were the aspects of “Improvement of the disease-related quality of life “(72.5%). In both cases, this common goal was only fully achieved for about one quarter of the patients (25.7%/24.1%), but partially achieved in more than half of the asked patients (53.2%/52.9%). Half of the patients reported that they achieved active participation in cancer treatment with integrative medicine. Around 50% partially achieved stabilization of the body, soul, and spirit, stress, disease management, improvement in cancer-related quality of life, and reduced the side effects of conventional cancer therapies. The success of integrative therapy was independent of age, BMI, family status, children, level of education, insurance type, alcohol and tobacco consumption, sport, low-fat diet, daily fruit and vegetable servings, interest in diets, and previous use of diets.

**Conclusions and discussion:**

Using a standardized procedure in integrative medicine allows patients to receive high-quality care. The previous standard of living has no effect on the benefits of integrative medicine for the patient. The goals through the use of integrative medicine could be achieved by all patient groups. It is highly encouraged to incorporate counseling and evidence-based integrative medicine into the clinical routines of cancer centers and adapt postgraduate medical education. Finally, the evidence base for the recommendations should also be strengthened by further research into the use of integrative medicine.

**Supplementary Information:**

The online version contains supplementary material available at 10.1007/s00404-025-08002-w.

## What does this study add to the clinical work

**Table:** 

The study clearly showed that integrative medicine can improve the quality of life of female cancer patients, regardless of their previous lifestyle or risk factors. Patients who were previously very healthy will benefit to the same extent as patients whose lifestyle was previously unhealthy, so access to integrative medicine should be open to all patient groups.	

## Introduction

### Cancer therapy and side effects

Over the past few years, thanks to the growing effectiveness of oncological therapies and improvements in screenings, long-term survival rates after breast cancer diagnosis have significantly improved [1]. In Europe, the 5-year survival rate for women diagnosed with breast cancer is approximately 88% [2]. New targeted therapies are being researched at a rapid pace and are finding application in an ever-changing therapeutic landscape [3, 4]. For gynecological cancer, new target treatments have been approved in the last few years and a high impact on the survival rate is expected for the years ahead [5, 6]. These good news have brought new challenges to the medical community. Moreover, with the complex, wide range of cancer treatments, including surgery, chemotherapy, endocrine therapy, and targeted therapies, the short- and long-term management of the side effects of these therapies have become an essential component of the patient treatment pathway [7–9]. Cancer survivors can experience acute and chronic side effects and are more likely to suffer from various secondary health problems, influencing health-related quality of life (HRQoL) and creating additional difficulty for the patients [10–14]. Approximately 26% of breast cancer patients report severe fatigue symptoms, which, in many instances, will be remaining for years after the completion of oncological therapy and are challenging to treat [15, 16]. On the other hand conditions like chemotherapy-induced nausea and vomiting can be well-managed in most patients with multi-agent anti-emetic medications [17]. Management can also require a dose reduction, a modification in schedule, and the use of gabapentin for symptomatic treatment [18]. In addition, current research is looking at ways to prevent this condition, including tactile stimulation, cryotherapy, and acupuncture [19, 20].

It is increasingly recognized that the assessment of patient-reported outcomes in terms of toxicity and quality of life is an important component of oncology that can be used to improve personalized therapeutic decision-making [21, 22]. Especially endocrine treatment has a wide range of side effects for most patients. Vasomotor symptoms, a decrease in libido, and vaginal dryness are all frequent menopausal side-effects in younger women that may significantly influence their overall quality of life. Aromatase inhibitors may produce vaginal dryness and dyspareunia [23].

Moreover, aromatase inhibitors commonly cause arthralgia (joint pain), which are typically handled with nonsteroidal anti-inflammatory drugs (NSAIDs) and exercise; however, these approaches lack solid evidence [24, 25].

Hot flashes are a common side effect of endocrine treatment, with tamoxifen and ovarian suppression-based regimens considered especially potent hot-flash inducers [26].

### Integrative medicine

Cancer patients often complement their standard cancer therapy with acupuncture, meditation, herbs, and nutritional supplements. According to a meta-analysis of surveys, such treatments have gained popularity over the last few decades [27]. In general, more than half of the patients already use complementary and alternative medicine (CAM), and the trend is rising. Overall, users are more likely to be female, younger, more educated, and from more affluent socioeconomic backgrounds [28].

Various terms have been used to describe these therapies and their implementation into conventional care. For many years, CAM was the most widely recognized term, with “complementary” referring to treatments used in combination with conventional cancer therapy and “alternative” relating to therapies used in lieu of conventional cancer therapy [29]. If the various therapeutic approaches merely run in parallel without being coordinated with each other, possible interactions represent a risk [30].

In recent years, additional treatments such as acupuncture, mindfulness, yoga, and lifestyle counseling have been integrated into the major cancer centers [31, 32]. The phrase “integrative oncology” has become increasingly prominent as the word “integrative” better describes the kind of care and services at cancer treatment centers, where patients get these types of treatments in addition to conventional cancer treatments [30]. As defined by one definition of “integrative oncology”, the growing field is described as a “comprehensive, evidence-based approach to cancer treatment that engages all participants on all levels of their being and experience.” According to this definition, “integrative medicine” is the careful and deliberate integration of complementary medicine and conventional therapies in the patient’s best interest. It emphasizes different aspects of cancer patient treatment, such as focusing on the “body, mind, soul, and spirit” of the individual as well as the unique culture and natural environment. Specifically, the patient receives both conventional cancer therapy and the application of many different complementary procedures from a single source and in close coordination. This holistic aspect is summarized under the term “integrative medicine [33]. Integrative medicine assists patients in coping with the adverse effects of conventional cancer treatments or the disease itself and enhances their quality of life [34, 35].

Although integrative medicine is becoming more widely used, it is still not uniformly applied. This is due to the fact that there are no worldwide standards or norms for Complementary and Integrative Medicine (CIM.) Since 2021, there has been a guideline for complementary medicine in oncology in Germany (“S3-Leitlinie Komplementärmedizin in der Behandlung onkologischer Patienten”), which first presents the current therapeutic landscape at the highest level of evidence and aims, among other things, to attempt to harmonize the range of therapies available in Germany [36].

The main motivation for patients using CIM is the reduction of side effects [37].

In addition, patients focus on improving their mental health, reducing stress and anxiety and improving their overall quality of life [38]. Another aspect is that the patients, especially breast cancer patients, are interested in and benefit from becoming actively involved in the therapy [39]. Several obstacles still hamper the integration of CIM therapy with traditional cancer treatment. The majority of medical schools do not teach about CIM, and as a result, health care providers are unaware and contemptuous of these therapies. As a practical matter, many oncologists are still unable to consult on CIM treatments [40]. This creates a gap in care, and patients seek information outside the medical profession, which poses a safety risk to patients and their therapy [41, 42].

### Healthy lifestyle

The definition of a healthy lifestyle, in terms of diet and exercise, as well as the consumption of noxious substances, is not uniform in detail and has changed over the years. Nevertheless, there are some factors that define the current standard of a healthy lifestyle with sufficient evidence. These include diet, focus on conscious eating and proportion of vegetables [43]. Exercise in the sense of sporting activity, the body mass index, low alcohol consumption and nicotine abstinence [44, 45]. Much data already showed the impact of an active healthy lifestyle on cancer risk as well [46]. Therefore, in this study, we also focused on the mentioned lifestyle factors and specifically interrogated them.

## Methods

The study protocol was approved by the Ethics Committee of the Friedrich Alexander University Erlangen/Nuernberg on September 13, 2016 with reference number 255_16 B.

### Study design

The present study is a cross-sectional study conducted in the Special Consultation for Integrative Medicine (SIM) in the Women's Hospital of the University Hospital Erlangen. A baseline Integrative Medicine Questionnaire (IMed questionnaire) was collected from all patients at their initial visit to the Special Consultation for Integrative Medicine. From November 2016 to March 2017, a follow-up survey of patients was conducted on their visit to the special consultation for integrative medicine. The procedure of the SIM and the surveys is defined by a "Standard Operation Procedure" (SOP) and was validated successfully years ago [47]. The questionnaire can be found in the supplemental.

Data from the baseline questionnaire, follow-up interviews, and data from the hospital's own patient records on tumor disease and therapy were included in the analyses.

### Patients

The general inclusion criteria were a minimum age of 18 years, female gender, and breast or gynecologic cancer as well as the ability to understand the questionnaire. Written informed consent was available for all study participants.

From the establishment of the specialty consultation 255 patients attended SIM. Twenty-four were excluded because of lack of female gender, lack of any of the presumed gynecologic malignancies, or lack of understanding of the questions and ability to adequately respond. Twenty women died before the follow-up interview and seven patients did not give consent. For seven patients, the follow-up questionnaire was still pending at the time of the analysis. Thus, complete data were available for 197 patients.

**Fig. 1:**
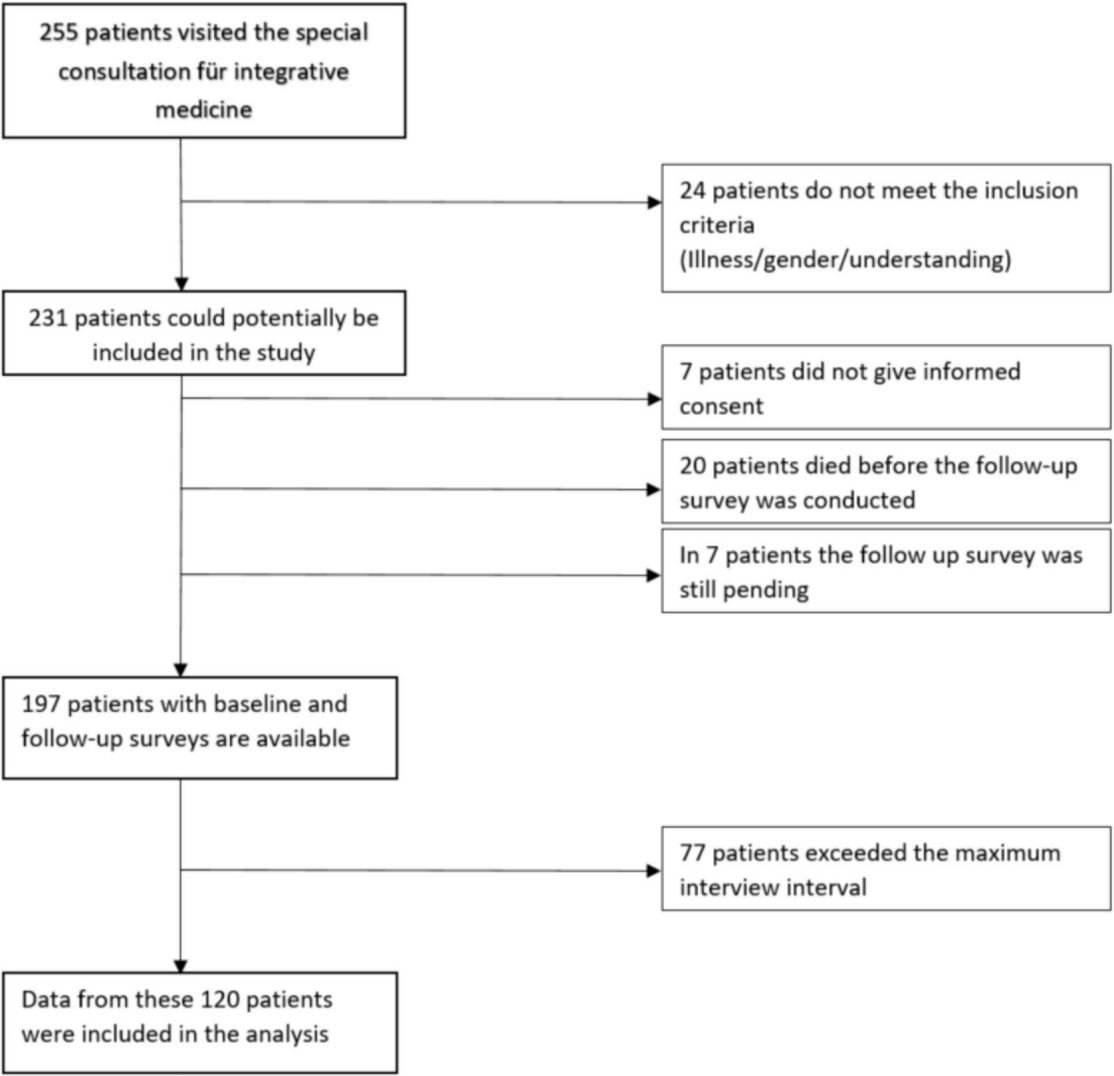
Flowchart of the IMed study: collective

Since the follow-up survey was initially carried out retrospectively for all patients who had previously attended the consultation, the time interval between visiting the consultation and answering the second questionnaire varied greatly at the beginning. To maintain comparability and to comply with the SOP of the consultation, a maximum interval of 6 months (+ 2 weeks) between the delivery of the therapy concept and the follow-up survey was In addition defined for this evaluation. As a result, 77 patients were excluded from the present analysis. In summary final analysis was carried out with 120 study participants, as also shown in Fig. 1.

At the first visit to the consultation, the standardized IMed basic questionnaire was handed out and completed by the patients on their own including information on general social factors, lifestyle, gynecological history and risk factors, and concomitant diseases, current tumor disease status, therapies received and current complaints as well as treatment goals. This was developed specifically for SIM and validated in terms of comprehensibility, complexity, comprehensiveness, and patient satisfaction [47]. This is available as a supplement and added to this publication.

The information serves as basis for creating an individual integrative therapy concept tailored to the patients’ needs.

The individual therapy concept is comprehensively explained during the personal second presentation in the consultation. The respective recommendations and open questions of the patient are discussed. The concept is also handed over to the patient in written form. The implementation of the therapy suggestions by the patients is done on their own responsibility.

The follow-up interview is conducted 3–6 months later to ensure a sufficient time interval for implementation of the therapy concept after it has been handed out at the second appointment. Interviews are conducted by telephone or in person at an appointment at the women's clinic. A separate follow-up questionnaire was also developed for this purpose. This contains 13 standardized questions, which address the complaints and therapy suggestions individually for each patient. Satisfaction with the therapy concept, its practicability and comprehensibility as well as with the atmosphere and organization of the consultation are also surveyed. The review of the implementation of the individual therapy recommendations as well as the follow-up of the stated complaints and therapy goals refer to the individual information from the basic questionnaire and the initial consultation. In addition, the follow-up includes questions about currently occurring complaints and new therapy goals if applicable.

### Data analysis and statistical evaluation

The data collected using the baseline and follow-up questionnaires were supplemented by information on tumor disease and therapy from the respective patient file and recorded in a database. Here, ambiguous and missing information as well as the answers "no information" were listed as "missing values" and were excluded from the analyses. A grading scale of 1–6, based on the German school grading system, was used for evaluation. In cases where the answer was between two grades or two grades were ticked, the lower grade was used for all evaluations. To be able to individually query complaints and suggestions for improvement for the consultation hours, free-text questions were used. For data analysis, these responses were grouped and both complaints and suggestions for improvement were named with an appropriate, consistent label. Multiple answers were possible for the questions on complaints, therapy goals, and suggestions for improvement.

Statistical analysis was performed using SPSS Statistics for Windows version 26 (IBM Corporation, Armonk in New York, USA). Mean, standard deviation, minimum, and maximum were calculated for age and BMI. The other characteristics regarding demographics, lifestyle, tumor disease, and therapy were analyzed based on their respective absolute and relative frequencies. For complaints and evaluation of consultation, the median and range of scores on complaint improvement and satisfaction were determined. Relative and absolute frequencies were calculated for therapy goals, evaluation of goal achievement, suggestions for SIM improvement, therapy recommendations, and their implementation. Therapy recommendations were considered successfully implemented as soon as they were carried out for at least 4 weeks. The number of therapy recommendations received per patient was examined using mean, standard deviation, minimum and maximum values.

## Results

### Patient’s characteristic, demographic and lifestyle factors

For a more detailed examination of the patient set, which comprises 120 female patients of the integrative medicine consultation, the age at the first visit of the SIM was first evaluated. The mean age was 51.6 ± 10.2 years. The majority of study participants could be assigned to the age group of 41–60 years (*n* = 77; 64.2%), 19 women (15.8%) were younger, and 24 women (20.0%) were older. Regarding the other demographic factors, most of them had insurance with a public health insurance company (*n* = 81; 68.4%), and in 37 cases (31.6%) there was private or supplementary insurance. Two patients (1.2%) did not provide any information. The most common educational qualification was a university degree (*n* = 53; 45.7%), followed by a Secondary School certificate degree (*n* = 44; 37.9%) and the Abitur (*n* = 19; 16.4%). Four patients (3.3%) did not provide information.

The collective includes patients with different tumor entities. These were first differentiated into breast carcinomas (*n* = 93; 77.5%) and gynecological carcinomas (*n* = 29; 24.2%). In the much smaller proportion of genital tumors, 18 (15.0% of the total) originated from the ovary, 7 (5.8% of the total) from the cervix, 3 (2.5% of the total) from the endometrium, and one (0.8% of the total) from the tube. One patient had both breast carcinoma and ovarian carcinoma present simultaneously. A look at the treatment situation showed that 103 (85.8%) patients received systemic therapy at the beginning. Of these, chemotherapy is the largest group, with 89 patients (86.4%). At the follow-up, approximately half of the patients were now undergoing systemic therapy, 62 (51.7%), with the chemotherapy group also being the largest group with 43 patients (69.4%). In addition to chemotherapy, questions were also asked about targeted or endocrine therapies. These were smaller groups at 16.5% and 37%, respectively. Surgery was named as the local therapy by 90 patients (75%), 35 (29.1%) stated radiotherapy. It should be noted that multiple answers were possible here.

The BMI value was calculated for each study participant; the mean value was 24.3 ± 4.3, the minimum was 15, and the maximum was 40. Accordingly, seven patients (5.8%) were assigned to the underweight category (BMI < 18.5). The largest proportion of 71 women (59.2%) were in the normal weight range (BMI 18.5–24.9), while 29 cases (24.2%) were overweight (BMI 25–29.9) and 8 cases (6.7%) were obese (BMI ≥ 30). Five patients (4.2%) did not make a statement. Table 1 shows all the patient characteristics examined.

**Table 1:** Patient characteristics (n = 120), showing absolute numbers, percentages and means

	*n*	%	MW (± SD)	Median (range)
Age			52.1 (10.1)	52.0 (25–74)
≤ 40	16	13.3		
41–60	77	64.2		
≥ 61	27	22.5		
Tumor location*				
Mamma	93	77.5		
Genital	29	24.2		
Ovaries	18	15.0		
Cervix	7	5.8		
Endometrium	3	2.5		
Tubes	1	0.8		
Baseline therapy*				
Systemic therapy	103	85.8		
Chemotherapy	89	86.4		
Endocrine therapy	17	16.5		
Targeted therapy	36	35.0		
Bisphosphonates	4	3.9		
Combined therapy	36	30.0		
Radiotherapy	35	29.1		
Completed radiotherapy	31	88.6		
Ongoing radiotherapy	4	11.4		
Surgery	90	75.0		
Baseline disease status				
Neoadjuvant	49	40.8		
Adjuvant	49	40.8		
Palliative	22	13.8		
Recurrence	12	10.0		
Follow-up therapy				
Systemic therapy	62	51.7		
Chemotherapy	43	69.4		
Endocrine therapy	14	22.6		
Targeted therapy	26	41.9		
Bisphosphonates	0	0		
Combined therapy	21	33.9		
Follow-up disease status				
Palliative, progression	8	7.5		
Palliative, stable disease	7	14.2		
Ongoing curative therapy	69	57,5		
Remission	27	22.5		
BMI^a^			24.3 (4.3)	24 (15–40)
Underweight (< 18.5)	7	5.8		
Normal weight (18.5–25)	71	59.2		
Overweight (25.1–30)	29	24.2		
Obesity (> 30)	8	6.7		
Not specified	5	4.2		
Marital status				
Married/in partnership	97	80.8		
Not married/not in partnership	21	17.5		
Not specified	2	1.7		
Children in household				
No children	36	30.0		
Children, but not in the household	51	42.5		
Children in household	32	26.7		
Not specified	1	0.8		
Education				
Secondary school	44	36.7		
High school diploma Abitur	51	15.8		
Study/University of Applied Sciences	32	44.2		
Not specified	4	3.3		
Health insurance				
Public health insurance	81	67.5		
Private health insurance	37	30.8		
Not specified	2	1.7		

*Multiple answers possible

^a^Missing values: 5

The common aspects of nutrition, sport and consumption were considered for the lifestyle factors. All details are listed in Table 2.

**Table 2:** Lifestyle factors and risk factors of patients at initial visit to SIM (*n* = 120)

	*n*	%
Alcohol consumption		
Never	68	56.7
Yes, 1–2 times per week	33	27.5
Yes, 3–6 times per week	8	6.7
Rather daily	4	3.3
Not specified	7	5.8
Smoking		
Never	71	59.2
Yes, in the past	38	31.7
Yes, currently	10	8.3
Not specified	1	0.8
Sports		
Never	25	20.8
Yes, about 1 h per week	31	25.8
Yes, about 2–4 h per week	42	35.0
Yes, more than 4 h per week	12	10.0
Not specified	10	8.3
Low fat diet		
No low fat diet	47	39.2
Predominantly low fat diet	56	46.7
I cannot judge	13	10.8
Not specified	4	3.3
Portions of fruit and vegetables per day		
Rather irregularly	7	5.8
1 portion 1 Portion	32	26.7
2–3 portions	66	55.0
4–5 portions	11	9.2
Not specified	4	3.3
Interest in dieting		
Never	29	24.2
Yes, already before the disease	60	50.0
Yes, since the disease	25	20.8
Not specified	6	5.0
Diets performed		
Yes	40	33.3
No	77	64.2
Not specified	3	2.5

The survey on other lifestyle factors revealed that 68 patients (60.2%) never consumed alcohol, that 33 patients (29.2%) consumed an alcoholic beverage about once or twice a week, 8 women (7.1%) three to six times a week, and 4 women (3.5%) rather every day. Seven (5.8%) of them did not provide information. Regarding smoking behavior, most female patients (*n* = 71; 59.7%) answered never having smoked. 38 study participants (31.9%) had smoked in the past. 10 women (8.4%) reported that they currently smoked. One person (0.8%) did not provide information about smoking behaviors.

When asked if they exercised regularly, 12 cases (10.9%) answered "Yes, more than 4 h a week." "Yes, 2–4 h a week" was mentioned in 42 cases (38.2%), which was the most frequent. 31 patients (28.2%) chose "Yes, about 1 h a week" and 25 times (22.7%) the answer "No, never" was given. 10 times (8.3%) no answer was provided. The answers to the question whether conscious attention was paid to a low-fat diet were answered in the affirmative by 56 women (48.3%), in the negative in 47 cases (40.5%) and could not be assessed by 13 participants (11.2%). Four (3.3%) did not give any information. Consumption of fruits and vegetables in single portions was reported as "Approximately 4–5 times per day" by 11 patients (9.2%), and "Approximately 2–3 times per day" by most patients (n = 66; 55.0%). "Approximately 1 time per day" was the response of 32 women (26.7%), and the fewest participants (n = 7; 5.8%) indicated "Rather irregularly." Four (3.3%) did not provide information. Half of the patients were already interested in a diet before the disease, 25 (20.8%) are now interested since the disease. So far, one-third (33.3%) of the patients had already followed a diet. Six patients (5.0%) did not provide information.

### Therapy goals and subjective goal achievement

Based on the patients' complaints and therapy goals, an individual therapy concept was created, which contained an average of 25 ± 6 recommendations. Individual aspects are complementary medical procedures. When used together and coordinated with further cancer therapy, as here in the context, this is generally referred to as integrative medicine. Only the effect of the entire recommendation on integrative medicine was examined; one method alone was not investigated.

The recommended integrative therapy methods were initially structured on the basis of the five pillars of Kneipp's natural healing methods. These include nutritional therapy and order therapy, e.g., a structured daily routine or relaxation exercises. In addition, methods of exercise therapy were recommended, such as regular walking, step goals, light endurance or strength training. Common recommendations from hydrotherapy included cold facial casts, alternating casts, or cold water applications to the extremities. Last, the pillar of phytotherapy is taken up with therapy suggestions, such as psyllium husks, lavender or the enzyme preparation Equinovo. Also recommended were other applications with sufficient evidence or experience. The patients were asked after the detailed explanation and a sufficient time interval in the follow-up whether they achieved their individual therapy goals.

In the baseline survey, study participants were able to indicate therapeutic goals they were pursuing with the use of integrative medicine. The subjective achievement of these therapy goals was evaluated during the follow-up survey. In addition to the reduction of side effects, goals for improving prognosis and maintaining quality of life were frequently mentioned. For the most part, the therapy goals could be at least "partially achieved". The goal of active cooperation could even be "fully achieved" in the majority of cases. In contrast, the delay of recurrences as well as the prolongation of lifetime by integrative medicine could not be adequately assessed in most cases (data shown in Supplementary Table 3).

Factors influencing the therapy goals in the treatment with integrative cancer.

Alleviating patients' concomitant symptoms was completely or partially achieved in 59.6% of patients, see Table 3 or . None of the socioeconomic factors mentioned above show a statistical significance in this context, see Supplementary Table 4. The previous lifestyle also had no influence on the achievement of the therapy goals, see Supplementary Table 5

For 90% of the patients, the reduction of side effects of conventional oncological therapy was one of the most frequently mentioned therapy goals. 73.9% of the patients were able to achieve this at least partially, see Table 3. Again, no relevant influencing factor was identifiable, neither in the general information, nor in the previous lifestyle, shown in Supplementary Table 6 and Table 7 .

The improvement of the quality of life, which is strongly influenced by the tumor disease, has been a desired therapeutic goal of integrative medicine for 72.5% of the patients. No significant influencing factor was found, see Supplementary Table 8 and Table 9.

An improvement in stress and disease management was sought by 55.8% of the patients surveyed. An influence variable on success, which occurred at least partially 68.6%, could not be shown significantly. Details are shown in Supplementary Table 10 and Table 11.

Improving body, mind and spirit in a holistic approach was the goal of a total of 65% of patients, see Table 3 or Here, too, no single influencing factor crystallized, which is shown in detail in Supplementary Table 12 and Table 13

As mentioned in the introduction one of the most important aspects of integrative medicine, active participation in the process of recovery was sought by 64.2% of patients in this study. The goal was achieved by 84.4%. Influences of the previous lifestyle or life circumstances could not be proven significantly, shown in Supplementary Table 14 and Table 15

When looking at the different therapy situations and types of therapy, no significant difference was found. When considering the influence of the disease status, there was only a change from the starting point in a few cases. Therefore, no significant influence could be presented. Data in detail not shown.

## Discussion

### Cancer entities

With 75.8%, breast carcinoma represents by far the most frequent localization of tumors in the present patient collective. With regard to tumor stage, no clear trend emerged in the present evaluations. Patients of all UICC stages sought out SIM. The majority of patients were in a curative therapy situation without the presence of distant metastases (77.5%). Especially breast carcinomas can often be diagnosed in early stages and can be treated well. In addition to the cure, the preservation of the quality of life and reduction of side effects is also a central aspect. Concordant observations were also shown in previous studies [48]. 23.7% of the participants had advanced disease and were, therefore, in a palliative situation (UICC stage IV). In this situation, the focus is no longer on a cure, but on stabilizing the disease, maintaining the best possible quality of life, and reducing symptoms. Due to the limited life expectancy and the unfavorable prognosis, the willingness to exhaust all available treatment options is high. This factor plays a major role in the use of integrative methods in advanced stages of the disease [49].

Although most patients with breast carcinomas and gynecologic carcinomas use local therapies, such as surgery or radiotherapy, only 12.5% of patients sought SIM in temporal relation to local therapy.

The majority of study participants were on systemic therapy (85.8%) at the time of the initial visit to SIM. In most cases, patients received chemotherapy alone (86.4.0%) or in combination with targeted therapy (30%), as well as endocrine therapies. The fact that patients undergoing systemic therapies, especially chemotherapy, show a high interest in integrative medicine is consistent with the results of other study groups [50]. These drug therapies can cause a variety of side effects and significantly reduce patients' quality of life. They have a major impact on the daily life and well-being of patients and are, therefore, often perceived as very burdensome. As already described, no significant differences were found when considering the type of therapy and the therapy situation. One possible explanation is certainly the low proportion of patients from the consultation who did not receive chemotherapy.

It is again important to emphasize that it was possible to give several answers at the same time. It is, therefore, much more difficult to show a possible difference between surgery, endocrine therapy, radiotherapy or chemotherapy. This is certainly a limitation of the evaluation. Further surveys of purely surgical patients, e.g., would be a possibility. However, this would probably have to take place outside of a special consultation for integrative medicine, as there seems to be little demand for this special consultation from this group, at least actively. When looking at the disease status, there was only a change from the starting point in a few cases. Therefore, no significant differences were found here either. A longer follow-up of years would generate further data here and could possibly reveal an influence.

### Lifestyle factors

The evaluations performed showed that the female patients of the integrative consultation mostly followed a healthy lifestyle.

The majority of women (57.4%) in our collective had a BMI in the normal weight range with a mean BMI value of 24.3 ± 4.4 kg/m^2^. In comparison, the mean BMI value of the total female population over 18 years of age in Germany was higher, at 25.1 kg/m^2^ according to the 2017 microcensus. The proportion of normal-weight women was lower in the female population at 53.6% with higher percentages of overweight and obese women compared with the study participants [51]. In each case, about 60%, and thus the majority of female patients surveyed, reported never drinking alcohol or never smoking. The vast majority exercised regularly and ate fruits and vegetables several times a day. With 48.3%, just under half of the study participants stated that they consciously paid attention to a low-fat diet.

In summary, the results indicate that the female patients in the collective maintain a very health-conscious lifestyle. Other studies also report associations between physical activity, healthy diet, a generally healthy lifestyle, and a clustered use of integrative methods [52].

### Therapy goals and influence on subjective goal achievement

Overall, carcinoma therapies often have considerable side effects, which represent an additional burden in addition to the serious diagnosis and the discomfort caused by the tumor disease itself. On median, most complaints were "somewhat improved" using integrative therapies.

To investigate the reasons for the use of integrative medicine among female cancer patients in more detail, the therapy goals were surveyed. The central goal of the use of integrative medicine was the reduction of side effects of conventional therapies. This was expressed by 92% of the study participants. In addition, about three quarters of all patients stated that the treatment goal was to delay the occurrence of recurrences or metastases and to improve the disease-related quality of life. Prolongation of lifespan, stabilization of body, mind and spirit, active cooperation in coping with the cancer, improvement of stress and disease management and alleviation of symptoms of the cancer were also frequently mentioned in the collective. This is consistent with findings from other studies and previous evaluations of SIM [14, 53, 54]. In the follow-up survey, the goals of using integrative therapies were predominantly assessed by the patients as "partially achieved". Active cooperation was even rated as "fully achieved" by 56% of the study participants with this goal. About a quarter of the women were subjectively able to fully achieve the therapy goals "reduction of side effects", "improvement of disease-related quality of life" and "alleviation of symptoms of cancer". The goals of "delaying recurrences or metastases" and "prolonging life" were less well assessed, so that the patients mostly rated the achievement of these goals as "don't know". As a rule, these are goals that usually cannot be assessed in the time frame of up to 6 months, but exist in the long term.

At the beginning of the study, one could have postulated the assumption that patients who were already more aware and healthier so far were more likely to achieve their goals. Or one might have thought the opposite that counseling by the integrative consultation would have less impact on patients with very healthy lifestyles and good resources from a socioeconomic perspective. However, this study has been able to show neither, gratifyingly for the patients but showed a positive effect on their goals through the use of integrative medicine regardless of status or lifestyle in recent years.

### Strengths and limitations of the study

This study is a cross-sectional study with retrospective data collection. Part of the study data has already been analyzed. In the meantime, further data was collected and the maximum period between the SIM visit and the follow-up interview was limited to 6 months.

The study has many strengths, but has some limitations. It can be positively emphasized that both breast carcinoma patients and genital carcinoma patients were included in the study. In the case of genital carcinoma patients, there is less data available on the use of integrative medicine, so that there is definitely a need for the evaluation of integrative medicine methods here. However, the number of study participants with genital cancers was lower compared to breast cancer patients. This reflects the frequencies of the individual carcinoma diseases in the overall population of Germany.

Recruitment and implementation took place in the SIM at the Erlangen Women's Hospital. This results in a restricted, clearly defined collective without a control group, which is why there is a risk of bias with regard to the study population. However, the visit of the integrative consultation was open to all patients, including persons in external treatment. The telephone or personal follow-up survey ensured that the questions were understood and answered correctly.

The response rate of the follow-up interviews was very high (85%), with 197 of 231 patients meeting the general inclusion criteria. Due to the limitation of the survey interval, a large number of patients (*n* = 77) were excluded, so that finally 120 of the questionnaires could be evaluated. This led in part to small sub-collectives in the evaluations.

The interview interval was inhomogeneous in the preliminary evaluations due to the initial retrospective recruitment of study participants. Meanwhile, this was done prospectively at initial SIM visit. The maximum time interval between the visit to the consultation and the follow-up survey was limited to a maximum of 6 months for the present evaluations. This increased the comparability and quality of the data. The aim was, for example, to improve recall on implementation of treatment recommendations or improvement of symptoms.

Furthermore, it is difficult to draw conclusions about the effectiveness of integrative methods in detail from the data, since only the concept as a whole was evaluated. On the other hand, integrative medicine aims to enable holistic, complementary treatment and not to apply individual measures in isolation. Moreover, these were subjective assessments by the study participants. Objective measurements, for example, of the benefit of the integrative concept in terms of improvement of complaints or quality of life are not available. Further studies with suitable measuring instruments and comparison with a control group without the use of integrative methods are required.

## Conclusion

It was clearly demonstrated that integrative medicine, i.e., the combination of conventional oncology with complementary medical procedures, brings benefits for every patient. It should be emphasized that a previous “healthy lifestyle” or even ‘bad habits’ are no indication of the future success of integrative medicine. Therefore, every patient should have access to integrative medicine, to holistically support female carcinoma patients with conventional therapies. To achieve this, integrative medicine should also play a role in the training of doctors and oncologists. The introduction of the S3 guideline can only be considered as a starting point; implementation for a broad collective of female patients would be essential. Furthermore, the data on the use of integrative medicine should be extended to further improve the holistic therapy of cancer patients. Here, a larger collective and an objectification of the data should be aimed at.

## Supplementary Information

Below is the link to the electronic supplementary material.

Supplementary file1 (PDF 275 KB)

Supplementary file2 (DOCX 55 KB)

## Data Availability

No datasets were generated or analysed during the current study.
