# Supplementary material for: Impact of risk and lifestyle factors on therapy goals in the treatment of breast cancer and gynecological cancer patients with integrative medicine
Source: Arch Gynecol Obstet. 2025 Apr 9;311(6):1683–95. doi: 10.1007/s00404-025-08002-w (PMC12055625; doi:10.1007/s00404-025-08002-w)
Supplement: Supplementary file 1 — Supplementary file1 (PDF 275 KB) [file 404_2025_8002_MOESM1_ESM.pdf]

Universitätsklinikum Erlangen

Fragebogen für Integrative Medizin

Patientinnenaufkleber

Datum: ______________________

Arzt: ______________________

Fragen nach aktueller Behandlungssituation, derzeitiger Krebstherapie, Beschwerdesymptomatik, Begleitmedikamenten und Zielen, die Sie sich von der Integrativen Medizin erhoffen

Bitte füllen Sie diesen Fragebogen aus, damit wir gemeinsam ein individuelles Behandlungskonzept für Integrative Medizin erstellen können! Die Fragen beziehen sich auf die gegenwärtige Situation. Zögern Sie nicht bei Unklarheiten Ihre behandelnde Ärztin oder Ihren behandelnden Arzt zu fragen.

**Frage 1:** Was ist der höchste Grad Ihrer Schulausbildung?	☐ Haupt-/Realschule<br>☐ Abitur<br>☐ Studium/Fachhochschule
**Frage 2:** Wie ist Ihr Familienstand?	☐ verheiratet / in Partnerschaft<br>☐ nicht verheiratet / nicht in Partnerschaft
**Frage 3:** Wie alt ist Ihr jüngstes Kind, welches in Ihrem Haushalt wohnt (falls zutreffend)?	☐ Ich habe keine Kinder.<br>☐ Kind(er) wohnt(-en) nicht mehr in meinem Haushalt.<br>☐ Jüngstes Kind im Haushalt ist ____ Jahre alt.
**Frage 4:** Welche Religionszugehörigkeit haben Sie?	☐ christlich ☐ andere<br>☐ muslimisch ☐ keine
**Frage 5:** Wie sind Sie krankenversichert?	☐ gesetzlich ☐ privat oder mit Krankenhaus-zusatzversicherung<br>☐ keine Angabe
**Frage 6:** Rauchen Sie oder haben Sie geraucht?	☐ Nein, niemals<br>☐ Ja, früher, ich habe vor _____ Jahren aufgehört.<br>☐ Ja, ich rauche momentan, und zwar ca. ________ Zigaretten am Tag
**Frage 7:** Wie groß und schwer sind Sie aktuell?	_______ cm groß und ________ kg schwer
**Frage 8:** Wie oft in der Woche trinken Sie ein alkoholisches Getränk?	☐ Nie<br>☐ 1-2mal pro Woche<br>☐ 3-6mal pro Woche<br>☐ Eher jeden Tag

©IMed Frauenklinik UK-ER

**Frage 9:** Wie oft waren Sie schwanger?	_____________ mal schwanger
**Frage 10:** Wie viele Kinder haben Sie geboren?	_____________ (Anzahl der Kinder)
**Frage 11:** Wie alt waren Sie bei der ersten Geburt?	_____________ (Alter bei erster Geburt)
**Frage 12:** In welchem Alter hatten Sie Ihre erste Periodenblutung?	_____________ (Alter bei erster Menstruation)
**Frage 13:** Haben Sie aktuell noch Ihre Periodenblutung?	☐ Nein<br>☐ Ja, regelmäßig<br>☐ Ja, aber unregelmäßig<br><br>Letzte Menstruation? _____________(Tag/Monat/Jahr)
**Frage 14:** Haben Sie Hormone während oder nach den Wechseljahren als Tablette, Pflaster, Zäpfchen oder Creme eingenommen?	☐ Nein, nie<br>☐ Ja, bis zum Zeitpunkt meiner Krebserkrankung<br>☐ Ja, früher, aber nicht mehr zum Zeitpunkt meiner Krebserkrankung<br><br>Wenn ja, wie lange? _____________________ (Jahre)
**Frage 15:** Haben Sie vor den Wechseljahren Hormone zur Schwangerschaftsverhütung eingenommen (Tablette, Pflaster, Mirena®, Implanon®, NuvaRing®, 3-Monats-Spritze etc.?	☐ Nein, nie<br>☐ Ja, bis zum Zeitpunkt meiner Krebserkrankung<br>☐ Ja, früher, aber nicht mehr zum Zeitpunkt meiner Krebserkrankung<br><br>Wenn ja, wie lange? _____________________ (Jahre)
**Frage 16:** Treiben Sie regelmäßig Sport?	☐ Nein, niemals<br>☐ Ja, ca. 1 Stunde in der Woche<br>☐ Ja, ca. 2-4 Stunden in der Woche<br>☐ Ja, mehr als 4 Stunden in der Woche
**Frage 17:** Wie oft pro Tag essen Sie Obst oder Gemüse in einzelnen Portionen?	☐ Ich esse Obst und/oder Gemüse eher unregelmäßig<br>☐ Ich esse Obst und/oder Gemüse ca. 1 mal pro Tag<br>☐ Ich esse Obst und/oder Gemüse ca. 2 - 3mal pro Tag<br>☐ Ich esse Obst und/oder Gemüse ca. 4 - 5mal pro Tag
**Frage 18:** Achten Sie bewusst auf eine fettarme Ernährung?	☐ Nein<br>☐ Ja, ich ernähre mich vorwiegend fettarm<br>☐ Kann ich nicht beurteilen
**Frage 19:** Interessieren Sie sich für Diäten und Ernährungsprogramme?	☐ Nein, nie<br>☐ Ja, schon vor meiner Krebserkrankung<br>☐ Ja, seit ich Krebs habe
**Frage 20:** Haben Sie bereits eine Diät mit dem Ziel der Gewichtsabnahme gemacht?	☐ Nein<br>☐ Ja
**Frage 21:** Würden Sie sich von einer Diät oder Ernährungsumstellung eine bessere Lebensqualität erhoffen?	☐ Ja<br>☐ Nein<br>☐ Weiß ich nicht

**Frage 22:** Würden Sie sich von einer Diät oder Ernährungsumstellung eine bessere Krebsheilung erhoffen?	☐ Ja<br>☐ Nein<br>☐ Weiß ich nicht.
**Frage 23:** Haben Sie bereits diätetische Lebensmittel angewandt?	☐ Nein<br>☐ Ja *(bitte geben Sie an welche)*

-
-
-
-

**Frage 24:** Ist bei Ihnen ein Diabetes mellitus („Zuckerkrankheit“) bekannt?	☐ Ja<br>☐ Nein<br>☐ Weiß ich nicht
**Frage 25:** Interessieren Sie sich für Integrative (früher komplementäre und alternative = unterstützende, ergänzende) Medizin?	☐ Nein, nie<br>☐ Ja, schon vor meiner Krebserkrankung<br>☐ Ja, seit dem Zeitpunkt meiner Krebserkrankung
**Frage 26:** Haben Sie sich schon über Methoden der integrativen Therapien informiert?	☐ Ja, wie viel?_____ (1 = etwas, 2 = mittel, 3 = ausführlich)<br>☐ Nein.<br>☐ Weiß ich nicht.
**Frage 27:** Gibt es in der Familie Krebserkrankungen?	☐ Nein<br>☐ Ja<br>*Bitte geben Sie an, wer betroffen ist und welche Krebserkrankung vorliegt!*<br>__________<br>__________<br>__________

Frage 28: Welche Krebserkrankung haben Sie?

☐ Brustkrebs	festgestellt am: ________________________(Monat/Jahr)
☐ Eierstockkrebs	festgestellt am: ________________________(Monat/Jahr)
☐ Gebärmutterhalskrebs	festgestellt am: ________________________(Monat/Jahr)
☐ Gebärmutterschleimhautkrebs	festgestellt am: ________________________(Monat/Jahr)
☐ Eileiterkarzinom	festgestellt am: ________________________(Monat/Jahr)
☐ Sarkom	festgestellt am: ________________________(Monat/Jahr)
☐ Granulosazelltumor	festgestellt am: ________________________(Monat/Jahr)
☐ Sonstiges: _________________________	festgestellt am: ________________________(Monat/Jahr)

©IMed Frauenklinik UK-ER

Frage 29: Haben Sie Metastasen?

☐ Ja ☐ Nein ☐ Weiß nicht

Wenn Sie keine Metastasen haben überspringen Sie bitte die Frage 30 und machen Sie bei Frage 31 weiter.

Frage 30: Falls Sie Frage 29 mit „Ja“ beantwortet haben, wo haben Sie die Metastasen? (Mehrfachantworten möglich)

☐ Knochen	festgestellt am: ____________ (Monat/Jahr)
☐ Leber	festgestellt am: ____________ (Monat/Jahr)
☐ Lunge	festgestellt am: ____________ (Monat/Jahr)
☐ Gehirn	festgestellt am: ____________ (Monat/Jahr)
☐ Lymphknoten	festgestellt am: ____________ (Monat/Jahr)
☐ Lungenfell (Pleuraerguss, Pleurakarzinose)	festgestellt am: ____________ (Monat/Jahr)
☐ Bauchfell (Aszites, Peritonealkarzinose)	festgestellt am: ____________ (Monat/Jahr)
☐ Weiß ich nicht	festgestellt am: ____________ (Monat/Jahr)

Frage 31: Wurden Sie wegen Ihrer Krebserkrankung bereits operiert?

☐ Ja
☐ Nein, ist aber geplant
☐ Nein, ist auch nicht geplant
☐ Weiß nicht

Wenn ja, wann war die letzte Operation wegen Ihrer Krebserkrankung?

____________________ (Tag/Monat/Jahr)

Frage 32: Erhalten Sie aktuell eine Chemotherapie oder haben Sie in der Vergangenheit eine Chemotherapie erhalten?

☐ Ja, erhalte ich aktuell Welche? ____________________

Letzte Chemotherapie: ____________ (Monat/Jahr)

☐ Ja, habe ich erhalten Welche? ____________________

Letzte Chemotherapie: ____________ (Monat/Jahr)

☐ Nein
☐ Weiß nicht

Frage 33: Erhalten Sie aktuell eine Antihormontherapie oder haben Sie in der Vergangenheit eine Antihormontherapie erhalten (z.B. Tamoxifen (z.B. Nolvadex®, Tamoxifen-ratiopharm®), Letrozol (z.B. Femara®), Anastrozol (z.B. Arimidex®), Exemestan (z.B. Aromasin®), Fulvestrant (Faslodex®), GnRH-Spritzen (z.B. Enantone®, Trenantone®))?

☐ Ja, erhalte ich aktuell Welche? ____________________

Letzte Antihormontherapie: ____________ (Monat/Jahr)

☐ Ja, habe ich erhalten Welche? ____________________

Letzte Antihormontherapie: ____________ (Monat/Jahr)

☐ Nein
☐ Weiß nicht

©IMed Frauenklinik UK-ER

Frage 34: Erhalten Sie aktuell eine zielgerichtete Therapie oder haben Sie in der Vergangenheit eine zielgerichtete Therapie erhalten (z.B. Trastuzumab (Herceptin®), Pertuzumab (Perjeta®), Lapatinib (Tyverb®), Bevacizumab (Avastin®), Trastuzumab Emtansin (T-DM1, Kadcyla®), Everolimus (Afinitor®), Afatinib (BIBW 2992, Gilotrif®), Denosumab (Xgeva®))?

☐ Ja, erhalte ich aktuell — Welche? ____________________

Letzte zielgerichtete Therapie: ____________________ (Monat/Jahr)

☐ Ja, habe ich erhalten — Welche? ____________________

Letzte zielgerichtete Therapie: ____________________ (Monat/Jahr)

☐ Nein
☐ Weiß nicht

Frage 35: Erhalten Sie aktuell eine Bisphosphonattherapie oder haben Sie in der Vergangenheit eine Bisphosphonattherapie erhalten (z.B. Zoledronat (Zometa®), Ibandronat (Bondronat®), Alendronat (z.B. Fosamax®, Alendronsäure-ratiopharm®))?

☐ Ja, erhalte ich aktuell — Welche? ____________________

Letzte Bisphosphonattherapie: ____________________ (Monat/Jahr)

☐ Ja, habe ich erhalten — Welche? ____________________

Letzte Bisphosphonattherapie: ____________________ (Monat/Jahr)

☐ Nein
☐ Weiß nicht

Frage 36: Nehmen Sie aktuell an einer klinisch-onkologischen Studie teil oder haben Sie in der Vergangenheit an einer klinischen Studie teilgenommen?

☐ Ja, nehme aktuell teil — Welche? ____________________

Zeitraum der Studie: ____________________ (Monat/Jahr bis Monat/Jahr)

☐ Ja, habe teilgenommen — Welche? ____________________

Zeitraum der Studie: ____________________ (Monat/Jahr bis Monat/Jahr)

☐ Nein

☐ Weiß nicht

Frage 37: Erhalten Sie aktuell eine Strahlentherapie?

☐ Ja

☐ Nein

☐ Weiß nicht

Frage 38: Haben Sie in der Vergangenheit eine Strahlentherapie erhalten?

☐ Ja, vor weniger als 8 Wochen
☐ Ja, vor mehr als 8 Wochen
☐ Nein
☐ Weiß nicht

©IMed Frauenklinik UK-ER

Frage 39:

Welche Methoden der integrativen Therapien wenden Sie aktuell bereits an bzw. haben Sie früher angewandt?

1.) Medizinische Systeme (medical systems) (*Sie können auch mehrere Antworten ankreuzen, wenn Sie mehrere Methoden aktuell anwenden oder früher angewandt haben)*

• Anthroposophische Medizin<br>*(Geisteswissenschaftliche Medizin nach den Lehren von Rudolph Steiner)*	☐ ja, seit wann: ___________ (Monat/Jahr)<br>☐ früher, wie lange (Monate): _______	☐ nie<br>☐ weiß ich nicht
• Ayurveda<br>(traditionelle indische Heilkunst)	☐ ja, seit wann: ___________ (Monat/Jahr)<br>☐ früher, wie lange (Monate): _______	☐ nie<br>☐ weiß ich nicht
• Homöopathie	☐ ja, seit wann: ___________ (Monat/Jahr)<br>☐ früher, wie lange (Monate): _______	☐ nie<br>☐ weiß ich nicht
• Wassertherapie nach Sebastian Kneipp	☐ ja, seit wann: ___________ (Monat/Jahr)<br>☐ früher, wie lange (Monate): _______	☐ nie<br>☐ weiß ich nicht
• Ordnungstherapie nach Sebastian Kneipp	☐ ja, seit wann: ___________ (Monat/Jahr)<br>☐ früher, wie lange (Monate): _______	☐ nein<br>☐ weiß ich nicht
• Bewegungstherapie nach Sebastian Kneipp	☐ ja, seit wann: ___________ (Monat/Jahr)<br>☐ früher, wie lange (Monate): _______	☐ nein<br>☐ weiß ich nicht
• Pflanzentherapie nach Sebastian Kneipp	☐ ja, seit wann: ___________ (Monat/Jahr)<br>☐ früher, wie lange (Monate): _______	☐ nein<br>☐ weiß ich nicht
• Traditionelle chinesische Medizin<br>*(chinesische Arzneimitteltherapie, chinesische Akupunktur)*	☐ ja, seit wann: ___________ (Monat/Jahr)<br>☐ früher, wie lange (Monate): _______	☐ nie<br>☐ weiß ich nicht

2.) Psyche-Körper-Interventionen (mind-body) (*Sie können auch mehrere Antworten ankreuzen, wenn Sie mehrere Methoden aktuell anwenden oder früher angewandt haben)*

• Autogenes Training<br>*(von innen erzeugte Entspannungstechnik)*	☐ ja, seit wann: ___________ (Monat/Jahr)<br>☐ früher, wie lange (Monate): _______	☐ nie<br>☐ weiß ich nicht
• Biofeedback	☐ ja, seit wann: ___________ (Monat/Jahr)<br>☐ früher, wie lange (Monate): _______	☐ nie<br>☐ weiß ich nicht
• Gebet	☐ ja, seit wann: ___________ (Monat/Jahr)<br>☐ früher, wie lange (Monate): _______	☐ nie<br>☐ weiß ich nicht
• Hypnose	☐ ja, seit wann: ___________ (Monat/Jahr)<br>☐ früher, wie lange (Monate): _______	☐ nie<br>☐ weiß ich nicht
• Kunsttherapien<br>*(Mal-, Musik-, Tanztherapie)*	☐ ja, seit wann: ___________ (Monat/Jahr)<br>☐ früher, wie lange (Monate): _______	☐ nie<br>☐ weiß ich nicht
• Meditation	☐ ja, seit wann: ___________ (Monat/Jahr)<br>☐ früher, wie lange (Monate): _______	☐ nie<br>☐ weiß ich nicht
• Progressive Muskelrelaxation nach Jacobson	☐ ja, seit wann: ___________ (Monat/Jahr)<br>☐ früher, wie lange (Monate): _______	☐ nie<br>☐ weiß ich nicht

©IMed Frauenklinik UK-ER

• Qigong<br>(chinesische Meditationsmethode)	☐ ja, seit wann: ___________ (Monat/Jahr)<br>☐ früher, wie lange (Monate): _______	☐ nie<br>☐ weiß ich nicht
• Reiki<br>*(esotherische Praktik zur Selbstheilung)*	☐ ja, seit wann: ___________ (Monat/Jahr)<br>☐ früher, wie lange (Monate): _______	☐ nie<br>☐ weiß ich nicht
• Simonton-Methode<br>*(Stärkung der Selbstheilungskräfte durch psychoonkologische Konzepte)*	☐ ja, seit wann: ___________ (Monat/Jahr)<br>☐ früher, wie lange (Monate): _______	☐ nie<br>☐ weiß ich nicht
• Sophrologie<br>*(Entspannungstechnik zur Herstellung des Gleichgewichts von Körper und Geist)*	☐ ja, seit wann: ___________ (Monat/Jahr)<br>☐ früher, wie lange (Monate): _______	☐ nie<br>☐ weiß ich nicht
• Thai Chi<br>*(chinesische Kampfkunst)*	☐ ja, seit wann: ___________ (Monat/Jahr)<br>☐ früher, wie lange (Monate): _______	☐ nie<br>☐ weiß ich nicht
• Yoga und Hormonyoga	☐ ja, seit wann: ___________ (Monat/Jahr)<br>☐ früher, wie lange (Monate): _______	☐ nie<br>☐ weiß ich nicht

3.) Manipulative und Körper-Therapien (body based) (*Sie können auch mehrere Antworten ankreuzen, wenn Sie mehrere Methoden aktuell anwenden oder früher angewandt haben)*

• Akupunktur/Akupressur	☐ ja, seit wann: ___________ (Monat/Jahr)<br>☐ früher, wie lange (Monate): _______	☐ nie<br>☐ weiß ich nicht
• Chiropraxis/ Manuelle Therapie<br>(Behandlung zur Wiederherstellung der Beweglichkeit von Gelenken)	☐ ja, seit wann: ___________ (Monat/Jahr)<br>☐ früher, wie lange (Monate): _______	☐ nie<br>☐ weiß ich nicht
• Fußreflexzonenmassage	☐ ja, seit wann: ___________ (Monat/Jahr)<br>☐ früher, wie lange (Monate): _______	☐ nie<br>☐ weiß ich nicht
• Hydro- und Thermotherapie	☐ ja, seit wann: ___________ (Monat/Jahr)<br>☐ früher, wie lange (Monate): _______	☐ nie<br>☐ weiß ich nicht
• Massage und Lymphdrainage	☐ ja, seit wann: ___________ (Monat/Jahr)<br>☐ früher, wie lange (Monate): _______	☐ nie<br>☐ weiß ich nicht
• Neuraltherapie<br>*(Beeinflussung d. vegetativen Nervensystems durch Lokalanästhetika)*	☐ ja, seit wann: ___________ (Monat/Jahr)<br>☐ früher, wie lange (Monate): _______	☐ nie<br>☐ weiß ich nicht
• Rolfing<br>*(Bindegewebsmassage)*	☐ ja, seit wann: ___________ (Monat/Jahr)<br>☐ früher, wie lange (Monate): _______	☐ nie<br>☐ weiß ich nicht
• Osteopathie<br>*(Therapie von Funktionsstörungen des Bewegungsapparats)*	☐ ja, seit wann: ___________ (Monat/Jahr)<br>☐ früher, wie lange (Monate): _______	☐ nie<br>☐ weiß ich nicht
• Shiatsu<br>*(Kombination aus manuellen und energetischen Behandlungstechniken)*	☐ ja, seit wann: ___________ (Monat/Jahr)<br>☐ früher, wie lange (Monate): _______	☐ nie<br>☐ weiß ich nicht
• Sport/Bewegung	☐ ja, seit wann: ___________ (Monat/Jahr)<br>☐ früher, wie lange (Monate): _______	☐ nie<br>☐ weiß ich nicht

4.) Biologische Therapien (biological based) (*Sie können auch mehrere Antworten ankreuzen, wenn Sie mehrere Methoden aktuell anwenden oder früher angewandt haben)*

•	Ernährungsrichtlinien	☐ ja, seit wann: __________ (Monat/Jahr)<br>☐ früher, wie lange (Monate): _______	☐ nie<br>☐ weiß ich nicht
•	Immunstimulanzien und Krebsimmuntherapie<br>*(z.B. Zytokine, Wachstumsfaktoren, Thymus-Serum-Faktor, Faktor AF 2, Bacillus Calmette-Guèrin, Kehole Limpert Hemocyanin, Levamisol, Dimepranol, Inosin etc.)*	☐ ja, seit wann: __________ (Monat/Jahr)<br>☐ früher, wie lange (Monate): _______	☐ nie<br>☐ weiß ich nicht
•	Krebsdiät	☐ ja, seit wann: __________ (Monat/Jahr)<br>☐ früher, wie lange (Monate): _______	☐ nie<br>☐ weiß ich nicht
•	Misteltherapie	☐ ja, seit wann: __________ (Monat/Jahr)<br>☐ früher, wie lange (Monate): _______	☐ nie<br>☐ weiß ich nicht
•	Orthomolekulare Therapie	☐ ja, seit wann: __________ (Monat/Jahr)<br>☐ früher, wie lange (Monate): _______	☐ nie<br>☐ weiß ich nicht
•	Pflanzentherapie	☐ ja, seit wann: __________ (Monat/Jahr)<br>☐ früher, wie lange (Monate): _______	☐ nie<br>☐ weiß ich nicht
•	Probiotika	☐ ja, seit wann: __________ (Monat/Jahr)<br>☐ früher, wie lange (Monate): _______	☐ nie<br>☐ weiß ich nicht
•	Schüßler-Salze	☐ ja, seit wann: __________ (Monat/Jahr)<br>☐ früher, wie lange (Monate): _______	☐ nie<br>☐ weiß ich nicht
•	Vitamine<br>*(z.B. Vitamin C, D, E, etc.)*	☐ ja, seit wann: __________ (Monat/Jahr)<br>☐ früher, wie lange (Monate): _______	☐ nie<br>☐ weiß ich nicht
•	Spurenelemente<br>*(z.B. Zink, Selen, etc.)*	☐ ja, seit wann: __________ (Monat/Jahr)<br>☐ früher, wie lange (Monate): _______	☐ nie<br>☐ weiß ich nicht
•	Enzyme<br>*(z.B. Bromelain, Papain, Trypsin, Chymotrypsin, Phlogenzym, Wobenzym, Equizym MCA, etc.)*	☐ ja, seit wann: __________ (Monat/Jahr)<br>☐ früher, wie lange (Monate): _______	☐ nie<br>☐ weiß ich nicht

5.) Energetische Therapien (energy based) (*Sie können auch mehrere Antworten ankreuzen, wenn Sie mehrere Methoden aktuell anwenden oder früher angewandt haben)*

	Elektrotherapie<br>*(Stimulation mit elektrischem Strom)*	☐ ja, seit wann: __________ (Monat/Jahr)<br>☐ früher, wie lange (Monate): _______	☐ nie<br>☐ weiß ich nicht
•	Licht- und Heliotherapie<br>*(Einsatz von Licht und Sonne)*	☐ ja, seit wann: __________ (Monat/Jahr)<br>☐ früher, wie lange (Monate): _______	☐ nie<br>☐ weiß ich nicht

6.) Sonstiges (*Sie können auch mehrere Antworten ankreuzen, wenn Sie mehrere Methoden aktuell anwenden oder früher angewandt haben)*

•	Antihomotoxikologie<br>*(Besondere Art der modernen Homöopathie)*	☐ ja, seit wann: __________ (Monat/Jahr)<br>☐ früher, wie lange (Monate): _______	☐ nie<br>☐ weiß ich nicht

• Chronotherapie *(Behandlungsmethoden bei zirkadianen Rhythmusstörungen, Schlafhygiene)*	☐ ja, seit wann: ___________ (Monat/Jahr)<br>☐ früher, wie lange (Monate): _______	☐ nie<br>☐ weiß ich nicht
• Klimatherapie	☐ ja, seit wann: ___________ (Monat/Jahr)<br>☐ früher, wie lange (Monate): _______	☐ nie<br>☐ weiß ich nicht
• Ozon- und Sauerstofftherapie	☐ ja, seit wann: ___________ (Monat/Jahr)<br>☐ früher, wie lange (Monate): _______	☐ nie<br>☐ weiß ich nicht
• Sonstiges:________________	☐ ja, seit wann: ___________ (Monat/Jahr)<br>☐ früher, wie lange (Monate): _______	☐ nie<br>☐ weiß ich nicht
• Sonstiges:________________	☐ ja, seit wann: ___________ (Monat/Jahr)<br>☐ früher, wie lange (Monate): _______	☐ nie<br>☐ weiß ich nicht
• Sonstiges:________________	☐ ja, seit wann: ___________ (Monat/Jahr)<br>☐ früher, wie lange (Monate): _______	☐ nie<br>☐ weiß ich nicht
• Sonstiges:________________	☐ ja, seit wann: ___________ (Monat/Jahr)<br>☐ früher, wie lange (Monate): _______	☐ nie<br>☐ weiß ich nicht

Frage 40: Welche Beschwerden stehen für Sie aktuell im Vordergrund?

(Mehrere Antworten möglich! Bitte kreuzen Sie an!)

☐ Reduzierte Denkfähigkeit

☐ Fatigue/ Müdigkeit/ Antriebslosigkeit

☐ Schmerzen — Wo? ______________________________

☐ Wechseljahresbeschwerden

☐ Durchfall

☐ Verstopfung

☐ Depressive Stimmung

☐ Beeinträchtigte Sexualaktivität

©IMed Frauenklinik UK-ER

Frage 41: Bitte nennen Sie die Ihrer Ansicht nach derzeit fünf wichtigsten Beschwerden und wie sehr Sie sich durch diese beeinträchtigt fühlen. Bitte nennen Sie die Beschwerden, die Sie am meisten beeinträchtigen, zuerst und die anderen dann in absteigender Wichtigkeit.

1.____________________________ beeinträchtigt mich ☐ immer ☐ oft ☐ selten

☐ stark ☐ mittel ☐ schwach

2.____________________________ beeinträchtigt mich ☐ immer ☐ oft ☐ selten

☐ stark ☐ mittel ☐ schwach

3.____________________________ beeinträchtigt mich ☐ immer ☐ oft ☐ selten

☐ stark ☐ mittel ☐ schwach

4.____________________________ beeinträchtigt mich ☐ immer ☐ oft ☐ selten

☐ stark ☐ mittel ☐ schwach

5.____________________________ beeinträchtigt mich ☐ immer ☐ oft ☐ selten

☐ stark ☐ mittel ☐ schwach

Frage 42: Nehmen Sie zusätzlich weitere Medikamente für andere Erkrankungen ein?

☐ Nein

☐ Ja *(bitte geben Sie an welche)*

- Medikament 1 ____________________ (Name des Medikaments) ____________________ (Dosierung, Häufigkeit)
- Medikament 2 ____________________ (Name des Medikaments) ____________________ (Dosierung, Häufigkeit)
- Medikament 3 ____________________ (Name des Medikaments) ____________________ (Dosierung, Häufigkeit)
- Medikament 4 ____________________ (Name des Medikaments) ____________________ (Dosierung, Häufigkeit)
- Medikament 5 ____________________ (Name des Medikaments) ____________________ (Dosierung, Häufigkeit)
- Medikament 6 ____________________ (Name des Medikaments) ____________________ (Dosierung, Häufigkeit)
- Medikament 7 ____________________ (Name des Medikaments) ____________________ (Dosierung, Häufigkeit)
- Medikament 8 ____________________ (Name des Medikaments) ____________________ (Dosierung, Häufigkeit)

©IMed Frauenklinik UK-ER

Frage 43: Welche Ziele haben Sie mit der zusätzlichen Verwendung der Integrativen Medizin?

Welche Ziele wollen Sie sofort erreichen?

(Kreuzen Sie bitte folgend die kurzfristigen Ziele an, die Sie haben)

- ☐ Linderung von Symptomen der Krebserkrankung
- ☐ Reduktion von Nebenwirkungen der konventionellen (Krebs-)Therapien
- ☐ Verbesserung der krankheitsbezogenen Lebensqualität

- ☐ Verbesserung der Stress- und Krankheitsbewältigung
- ☐ Stabilisierung von Körper, Seele und Geist
- ☐ Aktive Mitarbeit zur Bewältigung der Krebserkrankung

- ☐ Verzögerung des Wiederauftretens der Erkrankung (Rezidiv) oder von Metastasen bei Krebserkrankungen
- ☐ Verlängerung der Lebenszeit bei Krebserkrankungen

- ☐ Sonstiges: ______________________________
- ☐ Sonstiges: ______________________________
- ☐ Sonstiges: ______________________________

Welche Ziele wollen Sie auf lange Sicht erreichen?

(*Kreuzen Sie bitte folgend die langfristigen Ziele an, die Sie haben)*

- ☐ Linderung von Symptomen der Krebserkrankung
- ☐ Reduktion von Nebenwirkungen der konventionellen (Krebs-)Therapien
- ☐ Verbesserung der krankheitsbezogenen Lebensqualität

- ☐ Verbesserung der Stress- und Krankheitsbewältigung
- ☐ Stabilisierung von Körper, Seele und Geist
- ☐ Aktive Mitarbeit zur Bewältigung der Krebserkrankung

- ☐ Verzögerung des Wiederauftretens der Erkrankung (Rezidiv) oder von Metastasen bei Krebserkrankungen
- ☐ Verlängerung der Lebenszeit bei Krebserkrankungen

- ☐ Sonstiges: ______________________________
- ☐ Sonstiges: ______________________________
- ☐ Sonstiges: ______________________________

______________________________ ______________________________

Unterschrift Patientin Unterschrift Ärztin bzw. Arzt

©IMed Frauenklinik UK-ER
