# Supplementary material for: Impact of risk and lifestyle factors on therapy goals in the treatment of breast cancer and gynecological cancer patients with integrative medicine
Source: Arch Gynecol Obstet. 2025 Apr 9;311(6):1683–95. doi: 10.1007/s00404-025-08002-w (PMC12055625; doi:10.1007/s00404-025-08002-w)
Supplement: Supplementary file 2 — Supplementary file2 (DOCX 55 KB) [file 404_2025_8002_MOESM2_ESM.docx]

# Supplementary:

Table 3 Subjective achievement of the most important individual therapy goals of the patients from the SIM (number of patients n = 120). For the evaluation of the achievement of the therapy goals, only patients who stated the therapy goal at the initial visit

| **Therapy goal** | **N (%)** | **Fully achieved**  **(grades 1–2)** | **Partially achieved**  **(grades 3–4)** | **Not reached**  **(grades 5–6)** | **Not specified** |
| --- | --- | --- | --- | --- | --- |
| Relief of symptoms of the tumor disease | 57 (47.5) | 13 (22.8) | 21 (36.8) | 1 (1.8) | 22 (39.6) |
| Reduction of side effects of conventional (cancer) therapies | 109 (90.8) | 28 (25.7) | 58 (53.2) | 6 (5.5) | 17 (15.6) |
| Improvement of the disease-related quality of life | 87 (72.5) | 21 (24.1) | 46 (52.9) | 5 (5.7) | 15 (17.2) |
| Improvement of stress and disease management | 67 (55.8) | 8 (11.9) | 38 (56.7) | 4 (6.0) | 17 (25.4) |
| Stabilization of body, soul and spirit | 78 (65.0) | 15 (19.2) | 44 (56.4) | 6 (7.7) | 13 (16.7) |
| Active participation in coping with cancer | 77 (64.2) | 39 (50.6) | 26 (33.8) | 2 (2.6) | 10 (13.0) |

Table 4 Factors influencing age, BMI, marital status, children in the household, education, and health insurance on the subjective therapy goal "alleviation of symptoms of the tumor disease"; only patients who had stated the therapy goal at the initial interview were considered (number of patients n = 35). Missing values were not included in the analysis

| **Influential factor** | **Therapy goal Fully achieved**  **(grades 1–2)**  **n** | **Therapy goal Partially achieved**  **(grades 3–4)**  **n** | **Therapy goal**  **Not achieved**  **(grades 5–6)**  **n** | ***p*** |
| --- | --- | --- | --- | --- |
| **Age** | | | | |
| ≤ 40 | 2 | 2 | 0 | *0.993^*^* |
| 41 – 60 | 6 | 14 | 0 |  |
| ≥ 60 | 5 | 5 | 1 |  |
| **BMI ^a^** | | | | |
| Underweight (<18.5) | 1 | 1 | 0 | *0.079^*^* |
| Normal weight (18.5–25) | 9 | 7 | 1 |  |
| Overweight (25.1–30) | 3 | 8 | 0 |  |
| Obesity (>30) | 0 | 4 | 0 |  |
| **Marital status** | | | | |
| Married/in partnership | 8 | 15 | 1 | *0,430^#^* |
| Not married/not in partnership | 5 | 6 | 0 |  |
| **Children in household** | | | | |
| No children | 6 | 7 | 0 | *0.619^#^* |
| Children, but not in the household | 5 | 5 | 1 |  |
| Children in household | 4 | 9 | 0 |  |
| **Education** | | | | |
| Secondary school | 5 | 10 | 1 | *0.797^#^* |
| High school diploma Abitur | 2 | 3 | 0 |  |
| Study/University of Applied Sciences | 5 | 8 | 0 |  |
| **Health insurance** | | | | |
| Public health insurance | 8 | 16 | 1 | *0.286^#^* |
| private health insurance | 5 | 5 | 0 |  |

* Spearman correlation ^#^Kruskal–Wallis test Significance level 0.05
^a^ = missing values: 1

Table 5 Factors influencing alcohol consumption, smoking, sport, low-fat diet, daily fruit and vegetable portions, interest in diets, and diets followed on the subjective therapy goal "alleviation of symptoms of tumor disease"; only patients who had stated the therapy goal at the initial interview were included (number of patients n = 35). Missing values were not included in the analysis

| **Influential factor** | **Therapy goal Fully achieved**  **(grades 1–2)n** | **Therapy goal Partially achieved**  **(grades 3–4)**  **n** | **Therapy goal**  **Not reached**  **(grades 5–6)**  **n** | ***p*** |
| --- | --- | --- | --- | --- |
| **Alcohol consumption** | | | | |
| Never | 6 | 11 | 1 | *0.813^#^* |
| Yes, 1–2 times per week | 5 | 8 | 0 |  |
| Yes, 3–6 times per week | 1 | 1 | 0 |  |
| **Smoking** | | | | |
| Never | 7 | 12 | 0 | *0.784^#^* |
| Yes, in the past | 6 | 8 | 1 |  |
| Yes, currently | 0 | 1 | 0 |  |
| **Sports (4 missing)** | | | | |
| Never | 4 | 6 | 1 | *0.506^#^* |
| Yes, about 1 h per week | 2 | 7 | 0 |  |
| Yes, about 2–4 h per week | 4 | 4 | 0 |  |
| Yes, more than 4 h per week | 2 | 1 | 0 |  |
| **Low fat diet** | | | | |
| No low fat diet | 4 | 9 | 0 | *0.509^#^* |
| Predominantly low fat diet | 9 | 10 | 1 |  |
| I cannot judge | 0 | 2 | 0 |  |
| **Portions of fruit and vegetables per day** | | | | |
| Rather irregularly | 0 | 2 | 0 | *0.150^#^* |
| 1 portion 1 Portion | 3 | 8 | 1 |  |
| 2–3 portions | 7 | 11 | 0 |  |
| 4–5 portions | 2 | 0 | 0 |  |
| **Interest in dieting^a^** | | | | |
| Never | 5 | 4 | 0 | 0.161^#^ |
| Yes, already before the disease | 2 | 11 | 0 |  |
| Yes, since the disease | 6 | 5 | 1 |  |
| **Diets performed** | | | | |
| Yes | 11 | 10 | 1 | 0.079^#^ |
| No | 2 | 11 | 0 |  |

* Spearman correlation ^#^Kruskal–Wallis test Significance level 0.05

^a^ = missing values: 1

^b^ = missing values: 2

^c^ = missing values: 4

Table 6 Factors influencing age, BMI, marital status, children in the household, education, and health insurance on the subjective therapy goal "reduction of side effects of conventional (cancer) therapies"; only patients who had stated the therapy goal at baseline were included (number of patients n = 92). Missing values were not included in the analysis

| **Influential factor** | **Therapy goal Fully achieved**  **(grades 1–2)n** | **Therapy goal Partially achieved**  **(grades 3–4)**  **n** | **Therapy goal**  **Not achieved**  **(grades 5–6)**  **n** | ***p*** |
| --- | --- | --- | --- | --- |
| **Age** | | | | |
| ≤ 40 | 5 | 8 | 1 | *0.744^*^* |
| 41 – 60 | 16 | 40 | 2 |  |
| ≥ 60 | 7 | 10 | 3 |  |
| **BMI ^c^** | | | | |
| Underweight (<18.5) | 2 | 4 | 0 | *0.551^*^* |
| Normal weight (18.5–25) | 18 | 34 | 4 |  |
| Overweight (25.1–30) | 6 | 12 | 2 |  |
| Obesity (>30) | 1 | 5 | 0 |  |
| **Marital status ^a^** | | | | |
| Married/in partnership | 22 | 47 | 5 | *0.451^#^* |
| Not married/not in partnership | 6 | 11 | 0 |  |
| **Children in household ^a^** | | | | |
| No children | 11 | 14 | 2 | *0.577^#^* |
| Children, but not in the household | 7 | 13 | 2 |  |
| Children in household | 10 | 31 | 1 |  |
| **Education ^b^** | | | | |
| Secondary school | 11 | 19 | 2 | *0.885^#^* |
| High school diploma Abitur | 3 | 10 | 0 |  |
| Study/University of Applied Sciences | 13 | 28 | 3 |  |
| **Health insurance** | | | | |
| Public health insurance | 17 | 43 | 5 | *0.146^#^* |
| private health insurance | 11 | 15 | 1 |  |

* Spearman correlation ^#^Kruskal–Wallis test Significance level 0.05
^a^ = missing values: 1

^b^ = missing values: 3

^c^ = missing values: 4

Table 7 Factors influencing alcohol consumption, smoking, sport, low-fat diet, daily fruit and vegetable portions, interest in diets, and diets carried out on the subjective therapy goal "reduction of side effects of conventional (cancer) therapies"; only patients who had stated the therapy goal at the initial interview were included (number of patients n = 92). Missing values were not included in the analysis

| **Influential factor** | **Therapy goal Fully achieved**  **(grades 1–2)n** | **Therapy goal Partially achieved**  **(grades 3–4)**  **n** | **Therapy goal**  **Not reached**  **(grades 5–6)**  **n** | ***p*** |
| --- | --- | --- | --- | --- |
| **Alcohol consumption ^d^** | | | | |
| Never | 13 | 36 | 4 | *0.331^#^* |
| Yes, 1–2 times per week | 8 | 16 | 0 |  |
| Yes, 3–6 times per week | 3 | 3 | 1 |  |
| Rather daily | 2 | 1 | 0 |  |
| **Smoking ^a^** | | | | |
| Never | 15 | 35 | 2 | *0.988^#^* |
| Yes, in the past | 10 | 19 | 2 |  |
| Yes, currently | 3 | 4 | 1 |  |
| **Sports ^e^** | | | | |
| Never | 4 | 13 | 2 | *0.628^#^* |
| Yes, about 1 h per week | 6 | 17 | 0 |  |
| Yes, about 2–4 h per week | 12 | 18 | 2 |  |
| Yes, more than 4 h per week | 4 | 6 | 1 |  |
| **Low fat diet ^a^** | | | | |
| No low fat diet | 10 | 25 | 3 | *0.524^#^* |
| Predominantly low fat diet | 14 | 27 | 2 |  |
| I cannot judge | 4 | 6 | 0 |  |
| **Portions of fruit and vegetables per day ^b^** | | | | |
| Rather irregularly | 2 | 2 | 1 | *0,087^#^* |
| 1 portion 1 Portion | 3 | 18 | 3 |  |
| 2–3 portions | 19 | 31 | 1 |  |
| 4–5 portions | 3 | 7 | 0 |  |
| **Interest in dieting ^c^** | | | | |
| Never | 11 | 14 | 2 | *0.615^#^* |
| Yes, already before the disease | 11 | 29 | 2 |  |
| Yes, since the disease | 6 | 13 | 1 |  |
| **Diets performed ^b^** | | | | |
| Yes | 18 | 43 | 4 | *0.257^#^* |
| No | 10 | 14 | 1 |  |

* Spearman correlation ^#^Kruskal–Wallis test Significance level 0.05

^a^ = missing values: 1

^b^ = missing values: 2

^c^ = missing values: 3

^d^ = missing values: 5

^e^ = missing values: 7

Table 8 Factors influencing age, BMI, marital status, children in the household, education, and health insurance on the subjective therapy goal "improvement of disease-related quality of life"; only patients who had stated the therapy goal at baseline were included (number of patients n = 72). Missing values were not included in the analysis

| **Influential factor** | **Therapy goal Fully achieved**  **(grades 1–2)**  **n** | **Therapy goal Partially achieved**  **(grades 3–4)**  **n** | **Therapy goal**  **Not achieved**  **(grades 5–6)**  **n** | ***p*** |
| --- | --- | --- | --- | --- |
| **Age** | | | | |
| ≤ 40 | 4 | 4 | 1 | *0.241^*^* |
| 41 – 60 | 14 | 28 | 3 |  |
| ≥ 60 | 3 | 14 | 1 |  |
| **BMI ^b^** | | | | |
| Underweight (<18.5) | 2 | 3 | 0 | *0.998^*^* |
| Normal weight (18.5–25) | 10 | 25 | 3 |  |
| Overweight (25.1–30) | 6 | 14 | 2 |  |
| Obesity (>30) | 2 | 3 | 0 |  |
| **Marital status ^a^** | | | | |
| Married/in partnership | 14 | 38 | 4 | *0.153^#^* |
| Not married/not in partnership | 7 | 7 | 1 |  |
| **Children in household ^a^** | | | | |
| No children | 9 | 14 | 1 | *0.373^#^* |
| Children, but not in the household | 5 | 14 | 3 |  |
| Children in household | 7 | 17 | 1 |  |
| **Education ^c^** | | | | |
| Secondary school | 6 | 15 | 3 | *0.531^#^* |
| High school diploma Abitur | 5 | 10 | 0 |  |
| Study/University of Applied Sciences | 10 | 17 | 2 |  |
| **Health insurance ^a^** | | | | |
| Public health insurance | 15 | 33 | 4 | *0.748^#^* |
| Private health insurance | 6 | 12 | 1 |  |

* Spearman correlation ^#^Kruskal–Wallis test Significance level 0.05
^a^ = missing values: 1

^b^ = missing values: 2

^c^ = missing values: 4

Table 9 Factors influencing alcohol consumption, smoking, sport, low-fat diet, daily fruit and vegetable portions, interest in diets, and diets carried out on the subjective therapy goal "improvement of disease-related quality of life"; only patients who had stated the therapy goal at the initial interview were included (number of patients n = 72). Missing values were not included in the analysis

| **Influential factor** | **Therapy goal Fully achieved**  **(grades 1–2)**  **n** | **Therapy goal Partially achieved**  **(grades 3–4)**  **n** | **Therapy goal**  **Not achieved**  **(grades 5–6)**  **n** | ***p*** |
| --- | --- | --- | --- | --- |
| **Alcohol consumption ^d^** | | | | |
| Never | 11 | 24 | 5 | *0.546^#^* |
| Yes, 1–2 times per week | 7 | 12 | 0 |  |
| Yes, 3–6 times per week | 1 | 5 | 0 |  |
| Rather daily | 0 | 2 | 0 |  |
| **Smoking ^a^** | | | | |
| Never | 14 | 22 | 3 | *0.474^#^* |
| Yes, in the past | 6 | 19 | 1 |  |
| Yes, currently | 1 | 4 | 1 |  |
| **Sports ^e^** | | | | |
| Never | 2 | 10 | 4 | *0.071^#^* |
| Yes, about 1 h per week | 8 | 10 | 1 |  |
| Yes, about 2–4 h per week | 7 | 16 | 0 |  |
| Yes, more than 4 h per week | 2 | 7 | 0 |  |
| **Low fat diet ^a^** | | | | |
| No low fat diet | 9 | 15 | 3 | *0.950^#^* |
| Predominantly low fat diet | 9 | 27 | 0 |  |
| I cannot judge | 3 | 3 | 2 |  |
| **Portions of fruit and vegetables per day ^b^** | | | | |
| Rather irregularly | 2 | 2 | 1 | *0.254^#^* |
| 1 portion 1 Portion | 2 | 15 | 2 |  |
| 2–3 portions | 14 | 24 | 2 |  |
| 4–5 portions | 2 | 4 | 0 |  |
| **Interest in dieting ^b^** | | | | |
| Never | 6 | 7 | 2 | *0.629^#^* |
| Yes, already before the disease | 9 | 28 | 2 |  |
| Yes, since the disease | 6 | 9 | 1 |  |
| **Diets performed ^b^** | | | | |
| Yes | 14 | 29 | 3 | *0.844^#^* |
| No | 7 | 15 | 2 |  |

* Spearman correlation ^#^Kruskal–Wallis test Significance level 0.05
^a^ = missing values: 1

^b^ = missing values: 2

^c^ = missing values: 5

Table 10 Factors influencing age, BMI, marital status, children in the household, education, and health insurance on the subjective therapy goal "improvement of stress and disease management"; only patients who had stated the therapy goal at baseline were included (number of patients n = 50). Missing values were not included in the analysis

| **Influential factor** | **Therapy goal Fully achieved**  **(grades 1–2)**  **n** | **Therapy goal Partially achieved**  **(grades 3–4)**  **n** | **Therapy goal**  **Not achieved**  **(grades 5–6)**  **n** | ***p*** |
| --- | --- | --- | --- | --- |
| **Age** | | | | |
| ≤ 40 | 2 | 5 | 1 | *0.561^*^* |
| 41 – 60 | 5 | 25 | 2 |  |
| ≥ 60 | 1 | 8 | 1 |  |
| **BMI ^a^** | | | | |
| Underweight (<18.5) | 1 | 2 | 0 | *0.404^*^* |
| Normal weight (18.5–25) | 5 | 21 | 2 |  |
| Overweight (25.1–30) | 1 | 10 | 2 |  |
| Obesity (>30) | 1 | 4 | 0 |  |
| **Marital status^a^** | | | | |
| Married/in partnership | 6 | 29 | 3 | *0.936^#^* |
| Not married/not in partnership | 2 | 8 | 1 |  |
| **Children in household ^a^** | | | | |
| No children | 1 | 13 | 2 | *0.358^#^* |
| Children, but not in the household | 4 | 8 | 2 |  |
| Children in household | 3 | 16 | 0 |  |
| **Education ^b^** | | | | |
| Secondary school | 2 | 10 | 2 | *0.596^#^* |
| High school diploma Abitur | 2 | 7 | 0 |  |
| Study/University of Applied Sciences | 4 | 19 | 2 |  |
| **Health insurance ^b^** | | | | |
| Public health insurance | 6 | 23 | 3 | *0.817^#^* |
| Private health insurance | 2 | 13 | 1 |  |

* Spearman correlation ^#^Kruskal–Wallis test Significance level 0.05
^a^ = missing values: 1

^b^ = missing values: 2

Table 11 Factors influencing alcohol consumption, smoking, sport, low-fat diet, daily fruit and vegetable portions, interest in diets, and diets carried out on the subjective therapy goal "improvement of stress and disease management"; only patients who had stated the therapy goal at the initial interview were included (number of patients n = 50). Missing values were not included in the analysis

| **Influential factor** | **Therapy goal Fully achieved**  **(grades 1–2)**  **n** | **Therapy goal Partially achieved**  **(grades 3–4)**  **n** | **Therapy goal**  **Not achieved**  **(grades 5–6)**  **n** | ***p*** |
| --- | --- | --- | --- | --- |
| **Alcohol consumption ^e^** | | | | |
| Never | 3 | 21 | 2 | *0.744^#^* |
| Yes, 1–2 times per week | 4 | 8 | 2 |  |
| Yes, 3–6 times per week | 1 | 2 | 0 |  |
| Rather daily | 0 | 2 | 0 |  |
| **Smoking ^a^** | | | | |
| Never | 5 | 20 | 1 | *0.310^#^* |
| Yes, in the past | 2 | 14 | 3 |  |
| Yes, currently | 1 | 3 | 0 |  |
| **Sports ^d^** | | | | |
| Never | 0 | 3 | 2 | *0.121* |
| Yes, about 1 h per week | 3 | 12 | 0 |  |
| Yes, about 2–4 h per week | 4 | 14 | 1 |  |
| Yes, more than 4 h per week | 1 | 6 | 0 |  |
| **Low fat diet ^b^** | | | | |
| No low fat diet | 3 | 14 | 3 | *0.630^#^* |
| Predominantly low fat diet | 3 | 19 | 0 |  |
| I cannot judge | 2 | 3 | 1 |  |
| **Portions of fruit and vegetables per day ^c^** | | | | |
| Rather irregularly | 0 | 2 | 1 | *0.489^#^* |
| 1 portion 1 Portion | 3 | 8 | 2 |  |
| 2–3 portions | 5 | 22 | 1 |  |
| 4–5 portions | 0 | 3 | 0 |  |
| **Interest in dieting ^c^** | | | | |
| Never | 3 | 3 | 1 | *0.308^#^* |
| Yes, already before the disease | 2 | 23 | 2 |  |
| Yes, since the disease | 3 | 9 | 1 |  |
| **Diets performed ^c^** | | | | |
| Yes | 6 | 22 | 2 | *0.384^#^* |
| No | 2 | 13 | 2 |  |

* Spearman correlation ^#^Kruskal–Wallis test Significance level 0.05
^a^ = missing values: 1

^b^ = missing values: 2

^c^ = missing values: 3

^d^ = missing values: 4

Table 12 Factors influencing age, BMI, marital status, children in the household, education, and health insurance on the subjective therapy goal "stabilization of body, mind, and spirit"; only patients who had stated the therapy goal at the initial interview were considered (number of patients n = 65). Missing values were not included in the analysis

| **Influential factor** | **Therapy goal Fully achieved**  **(grades 1–2)**  **n** | **Therapy goal Partially achieved**  **(grades 3–4)**  **n** | **Therapy goal**  **Not achieved**  **(grades 5–6)**  **n** | ***p*** |
| --- | --- | --- | --- | --- |
| **Age** | | | | |
| ≤ 40 | 6 | 22 | 2 | 0.403^*^ |
| 41 – 60 | 2 | 13 | 2 |  |
| ≥ 60 | 6 | 22 | 2 |  |
| **BMI ^b^** | | | | |
| Underweight (<18.5) | 1 | 2 | 1 | 0.737^*^ |
| Normal weight (18.5–25) | 8 | 25 | 3 |  |
| Overweight (25.1–30) | 6 | 12 | 2 |  |
| Obesity (>30) | 0 | 3 | 0 |  |
| **Marital status ^b^** | | | | |
| Married/in partnership | 11 | 35 | 5 | *0.781^#^* |
| Not married/not in partnership | 3 | 8 | 1 |  |
| **Children in household ^a^** | | | | |
| No children | 5 | 15 | 3 | *0.843^#^* |
| Children, but not in the household | 4 | 20 | 0 |  |
| Children in household | 6 | 8 | 3 |  |
| **Education ^c^** | | | | |
| Secondary school | 3 | 17 | 2 | *0.481^#^* |
| High school diploma Abitur | 4 | 6 | 1 |  |
| Study/University of Applied Sciences | 8 | 18 | 3 |  |
| **Health insurance ^a^** | | | | |
| Public health insurance | 10 | 30 | 5 | *0.534^#^* |
| Private health insurance | 5 | 13 | 1 |  |

* Spearman correlation ^#^Kruskal–Wallis test Significance level 0.05
^a^ = missing values: 1

^b^ = missing values: 2

^c^ = missing values: 3

Table 13 Factors influencing alcohol consumption, smoking, sport, low-fat diet, daily fruit and vegetable portions, interest in diets and diets carried out on the subjective therapy goal "stabilization of body, soul and spirit"; only patients who had stated the therapy goal at the initial interview were taken into account (number of patients n = 65). Missing values were not included in the analysis

| **Influential factor** | **Therapy goal Fully achieved**  **(grades 1–2)**  **n** | **Therapy goal Partially achieved**  **(grades 3–4)**  **n** | **Therapy goal**  **Not achieved**  **(grades 5–6)**  **n** | ***p*** |
| --- | --- | --- | --- | --- |
| **Alcohol consumption ^e^** | | | | |
| Never | 5 | 23 | 5 | *0.061^#^* |
| Yes, 1–2 times per week | 5 | 12 | 1 |  |
| Yes, 3–6 times per week | 1 | 5 | 0 |  |
| Rather daily | 2 | 0 | 0 |  |
| **Smoking ^a^** | | | | |
| Never | 7 | 27 | 3 | *0.657^#^* |
| Yes, in the past | 6 | 12 | 3 |  |
| Yes, currently | 2 | 4 | 0 |  |
| **Sports ^d^** | | | | |
| Never | 1 | 4 | 3 | *0.157^#^* |
| Yes, about 1 h per week | 4 | 15 | 1 |  |
| Yes, about 2–4 h per week | 6 | 17 | 2 |  |
| Yes, more than 4 h per week | 3 | 4 | 0 |  |
| **Low fat diet ^b^** | | | | |
| No low fat diet | 4 | 18 | 3 | *0.252^#^* |
| Predominantly low fat diet | 9 | 19 | 1 |  |
| I cannot judge | 2 | 4 | 2 |  |
| **Portions of fruit and vegetables per day ^b^** | | | | |
| Rather irregularly | 1 | 2 | 1 | *0.857^#^* |
| 1 portion 1 Portion | 2 | 14 | 1 |  |
| 2–3 portions | 10 | 22 | 4 |  |
| 4–5 portions | 1 | 4 | 0 |  |
| **Interest in dieting ^c^** | | | | |
| Never | 3 | 7 | 1 | *0.268^#^* |
| Yes, already before the disease | 6 | 25 | 4 |  |
| Yes, since the disease | 6 | 8 | 1 |  |
| **Diets performed ^b^** | | | | |
| Yes | 8 | 28 | 4 | *0.378^#^* |
| No | 7 | 13 | 2 |  |

* Spearman correlation ^#^Kruskal–Wallis test Significance level 0.05

^a^ = missing values: 1

^b^ = missing values: 3

^c^ = missing values: 4

^d^ = missing values: 5

^e^ = missing values: 6

Table 14 Factors influencing age, BMI, marital status, children in the household, education, and health insurance on the subjective therapy goal "Active cooperation in coping with cancer"; only patients who had stated the therapy goal at the initial interview were considered (number of patients n = 67). Missing values were not included in the analysis

| **Influential factor** | **Therapy goal Fully achieved**  **(grades 1–2)**  **n** | **Therapy goal Partially achieved**  **(grades 3–4)**  **n** | **Therapy goal**  **Not achieved**  **(grades 5–6)**  **n** | ***p*** |
| --- | --- | --- | --- | --- |
| **Age** | | | | |
| ≤ 40 | 6 | 4 | 0 | 0.212^*^ |
| 41 – 60 | 28 | 17 | 0 |  |
| ≥ 60 | 5 | 5 | 2 |  |
| **BMI ^b^** | | | | |
| Underweight (<18.5) | 3 | 1 | 0 | 0.181^*^ |
| Normal weight (18.5–25) | 23 | 12 | 1 |  |
| Overweight (25.1–30) | 9 | 9 | 1 |  |
| Obesity (>30) | 3 | 3 | 0 |  |
| **Marital status ^a^** | | | | |
| Married/in partnership | 30 | 22 | 1 | *0.391^#^* |
| Not married/not in partnership | 9 | 4 | 0 |  |
| **Children in household ^a^** | | | | |
| No children | 12 | 8 | 0 | *0.976^#^* |
| Children, but not in the household | 10 | 6 | 1 |  |
| Children in household | 17 | 12 | 0 |  |
| **Education ^b^** | | | | |
| Secondary school | 11 | 11 | 1 | *0.289^#^* |
| High school diploma Abitur | 8 | 4 | 0 |  |
| Study/University of Applied Sciences | 20 | 10 | 0 |  |
| **Health insurance ^b^** | | | | |
| Public health insurance | 26 | 20 | 2 | *0.213^#^* |
| Private health insurance | 12 | 5 | 0 |  |

* Spearman correlation ^#^Kruskal–Wallis test Significance level 0.05

^a^ = missing values: 1

^b^ = missing values: 2

Table 15: Factors influencing alcohol consumption, smoking, sport, low-fat diet, daily fruit and vegetable portions, interest in diets, and diets carried out on the subjective therapy goal "Active cooperation in coping with cancer"; only patients who had stated the therapy goal at the initial interview were included (number of patients n = 67). Missing values were not included in the analysis.

| **Influential factor** | **Therapy goal Fully achieved**  **(grades 1–2)**  **n** | **Therapy goal Partially achieved**  **(grades 3–4)**  **n** | **Therapy goal**  **Not reached achieved**  **(grades 5–6)**  **n** | ***p*** |
| --- | --- | --- | --- | --- |
| **Alcohol consumption ^b^** | | | | |
| Never | 22 | 14 | 1 | *0.383^#^* |
| Yes, 1–2 times per week | 12 | 7 | 0 |  |
| Yes, 3–6 times per week | 1 | 4 | 0 |  |
| Rather daily | 2 | 1 | 0 |  |
| **Smoking ^a^** | | | | |
| Never | 23 | 14 | 0 | *0.782^#^* |
| Yes, in the past | 13 | 9 | 1 |  |
| Yes, currently | 3 | 3 | 0 |  |
| **Sports ^c^** | | | | |
| Never | 6 | 5 | 1 | *0.629^#^* |
| Yes, about 1 h per week | 9 | 7 | 0 |  |
| Yes, about 2–4 h per week | 17 | 10 | 0 |  |
| Yes, more than 4 h per week | 6 | 2 | 0 |  |
| **Low fat diet ^a^** | | | | |
| No low fat diet | 13 | 13 | 0 | *0.463^#^* |
| Predominantly low fat diet | 21 | 9 | 1 |  |
| I cannot judge | 5 | 3 | 0 |  |
| **Portions of fruit and vegetables per day ^a^** | | | | |
| Rather irregularly | 1 | 2 | 0 | *0.459^#^* |
| 1 portion 1 Portion | 9 | 8 | 1 |  |
| 2–3 portions | 24 | 15 | 0 |  |
| 4–5 portions | 4 | 1 | 0 |  |
| **Interest in dieting ^b^** | | | | |
| Never | 7 | 4 | 0 | *0.951* |
| Yes, already before the disease | 23 | 15 | 0 |  |
| Yes, since the disease | 9 | 5 | 1 |  |
| **Diets performed ^a^** | | | | |
| Yes | 22 | 17 | 1 | *0.275^#^* |
| No | 17 | 8 | 0 |  |

* Spearman correlation ^#^Kruskal–Wallis test Significance level 0.05
^a^ = missing values: 2

^b^ = missing values: 3

^c^ = missing values: 4
